# Development and external validation of prognostic models to predict sudden and pump-failure death in patients with HFrEF from PARADIGM-HF and ATMOSPHERE

**DOI:** 10.1007/s00392-021-01888-x

**Published:** 2021-06-08

**Authors:** Li Shen, Brian L. Claggett, Pardeep S. Jhund, William T. Abraham, Akshay Suvas Desai, Kenneth Dickstein, Jianjian Gong, Lars V. Køber, Marty P. Lefkowitz, Jean L. Rouleau, Victor C. Shi, Karl Swedberg, Michael R. Zile, Scott D. Solomon, John J. V. McMurray

**Affiliations:** 1 Division of Medicine, Hangzhou Normal University, Hangzhou, China; 2 British Heart Foundation Cardiovascular Research Centre, University of Glasgow, 126 University Place, Glasgow, G12 8TA UK; 3 The Cardiovascular Division, Brigham and Women’s Hospital, Boston, MA USA; 4 The Division of Cardiovascular Medicine, Davis Heart and Lung Research Institute, Ohio State University, Columbus, USA; 5 Stavanger University Hospital, Stavanger, Norway; 6 The Institute of Internal Medicine, University of Bergen, Bergen, Norway; 7 Novartis Pharmaceutical Corporation, East Hanover, NJ USA; 8 Rigshospitalet Copenhagen University Hospital, Copenhagen, Denmark; 9 Institut de Cardiologie, Université de Montréal, Montreal, Canada; 10 Department of Molecular and Clinical Medicine, University of Gothenburg, Gothenburg, Sweden; 11 Department of Veterans Administration Medical Center, Medical University of South Carolina and RHJ, Charleston, USA

**Keywords:** Sudden death, Pump failure death, Model, Risk, Heart failure, Device

## Abstract

**Background:**

Sudden death (SD) and pump failure death (PFD) are the two leading causes of death in patients with heart failure and reduced ejection fraction (HFrEF).

**Objective:**

Identifying patients at higher risk for mode-specific death would allow better targeting of individual patients for relevant device and other therapies.

**Methods:**

We developed models in 7156 patients with HFrEF from the Prospective comparison of ARNI with ACEI to Determine Impact on Global Mortality and morbidity in Heart Failure (PARADIGM-HF) trial, using Fine-Gray regressions counting other deaths as competing risks. The derived models were externally validated in the Aliskiren Trial to Minimize Outcomes in Patients with Heart Failure (ATMOSPHERE) trial.

**Results:**

NYHA class and NT-proBNP were independent predictors for both modes of death. The SD model additionally included male sex, Asian or Black race, prior CABG or PCI, cancer history, MI history, treatment with LCZ696 vs. enalapril, QRS duration and ECG left ventricular hypertrophy. While LVEF, ischemic etiology, systolic blood pressure, HF duration, ECG bundle branch block, and serum albumin, chloride and creatinine were included in the PFD model. Model discrimination was good for SD and excellent for PFD with Harrell’s C of 0.67 and 0.78 after correction for optimism, respectively. The observed and predicted incidences were similar in each quartile of risk scores at 3 years in each model. The performance of both models remained robust in ATMOSPHERE.

**Conclusion:**

We developed and validated models which separately predict SD and PFD in patients with HFrEF. These models may help clinicians and patients consider therapies targeted at these modes of death.

**Trial registration number:**

PARADIGM-HF: ClinicalTrials.gov NCT01035255, ATMOSPHERE: ClinicalTrials.gov NCT00853658.

**Graphics abstract:**

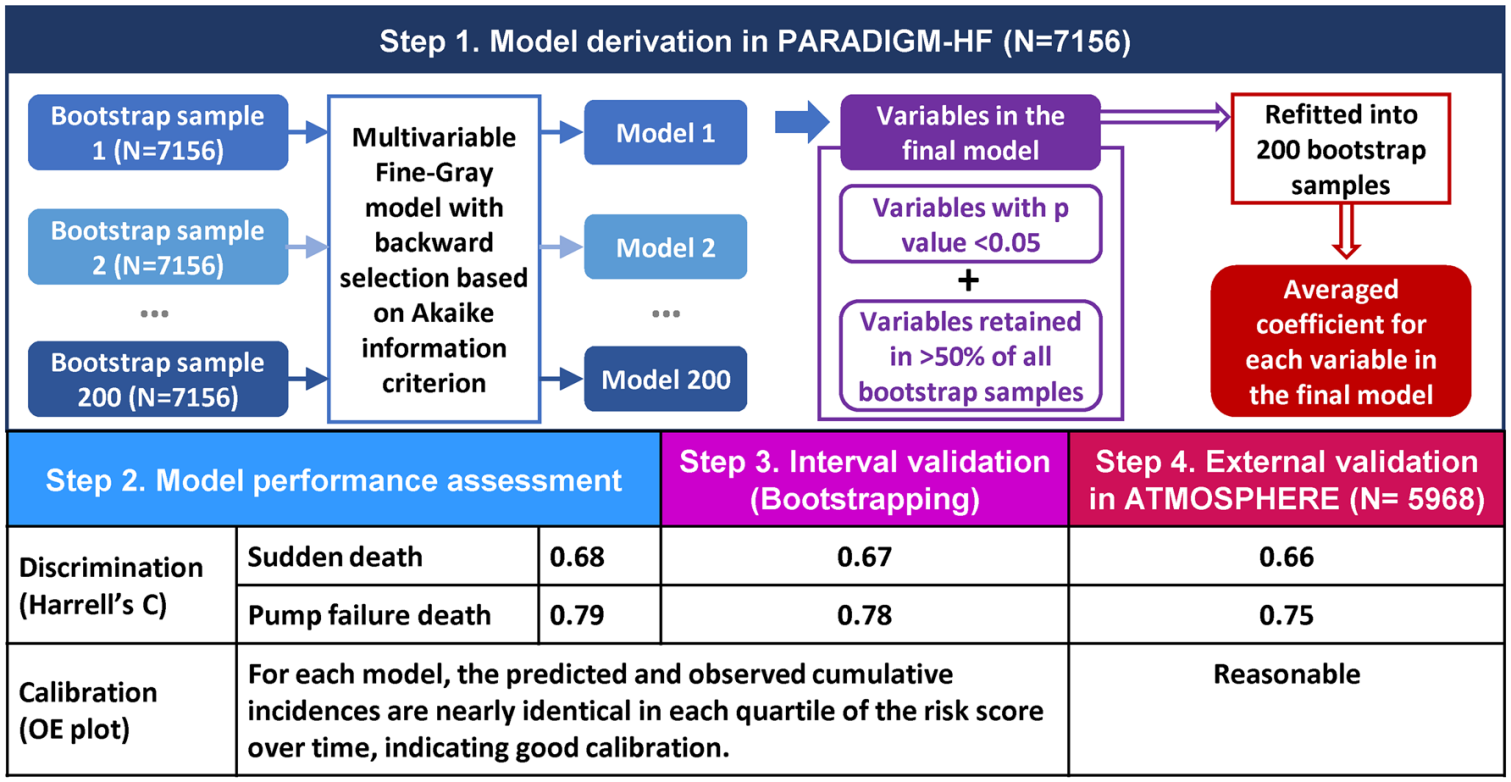

**Supplementary Information:**

The online version contains supplementary material available at 10.1007/s00392-021-01888-x.

## Introduction

Sudden death (SD) and pump failure death (PFD) are the predominant modes of death in patients with heart failure and reduced ejection fraction [1]. Quantifying an individual patient’s risk for mode-specific mortality can help with complex and difficult decisions about costly interventions, such as an implantable cardioverter defibrillator (ICD) or a left ventricular assist device, which are aimed at preventing specific causes of death [2].

One recent guideline suggests that risk calculators may be helpful in estimating an individual patient’s benefit/risk of an ICD implantation [3]. However, most existing risk models in patients with HF focus on predicting all-cause mortality [4–7]. Few models have been developed specifically for different modes of death and those that exist have some limitations. Statistically, several models are limited by having few events [8], most failed to take into account the prognostic influence of death from other causes [9], and crucially, none were externally validated [8–10], which is preferable for a model to be considered in clinical practice. Clinically, many models were built in cohorts in which few patients received modern evidence-based medications [10, 11]. In particular, the Seattle Heart Failure Model (SHFM) [4], designed to predict all-cause death, and which has also been shown to predict SD and PFD with good performance, was developed before the widespread use of beta-blockers and mineralocorticoid receptor antagonists (MRAs) [12]. Very recently, based on the same population, the authors developed the Seattle Proportional Risk Model (SPRM) to predict the proportion of deaths due to SD rather than the absolute risk [13]. It is unclear whether these models still perform well when applied to a contemporary cohort and, as recently demonstrated, the risk of sudden death has declined in parallel with improvements in medical therapy [14].

Theoretically, SD and PFD are two types of death with distinct risk profiles, and it is of interest to understand the potential association between different prognostic variables and each mode of death, especially in a single cohort and accounting for the competing risk of death from other causes.

The aims of this study were to develop and validate prognostic models separately for SD and PFD in patients with HFrEF, to compare the prognostic profiles of these modes of death, and to validate the SHFM and SPRM using the contemporary cohorts from the Prospective Comparison of ARNI with ACEI to Determine Impact on Global Mortality and Morbidity in Heart Failure Trial (PARADIGM-HF) [15] and the Aliskiren Trial to Minimize Outcomes in Patients with Heart Failure (ATMOSPHERE) [16].

## Methods

### Study population

This study consisted of a derivation cohort of patients in PARADIGM-HF and a validation cohort in ATMOSPHERE. Patients having an ICD or cardiac resynchronization therapy with a defibrillator (CRT-D) were excluded as these devices selectively reduce the risk of one of the two modes of death of interest. The design and results of both studies are published [15, 16].

Briefly, PARADIGM-HF evaluated the effect of LCZ696 with enalapril in 8399 patients with a left ventricular ejection fraction (LVEF) ≤ 40% (changed to ≤ 35% by amendment) and NYHA class II-IV HF, in addition to recommended treatment including an angiotensin converter enzyme (ACE) inhibitor or angiotensin receptor blocker (ARB) and a beta-blocker (unless contraindicated) and a MRA (if indicated). Patients were required to have a plasma B-type natriuretic peptide (BNP) ≥ 150 pg/ml (or N-terminal pro-BNP [NT-proBNP] ≥ 600 pg/ml), or a BNP ≥ 100 pg/ml (or NT-proBNP ≥ 400 pg/ml) and a HF hospitalization within the past 12 months. The key exclusion criteria included intolerance of ACE inhibitors or ARBs, a history of angioedema, symptomatic hypotension, a systolic blood pressure (SBP) < 100 mmHg at screening (< 95 mmHg at randomization), an estimated glomerular filtration rate (eGFR) < 30 ml/min/1.73m^2^, and a serum potassium level > 5.2 mmol/L at screening (> 5.4 mmol/L at randomization). Patients were accrued from December 8, 2009, through November 23, 2012 from 1043 centers in 47 countries, and the follow-up ended on March 31, 2014. The median follow-up was 27 months.

ATMOSPHERE compared aliskiren monotherapy and aliskiren/enalapril combination therapy with enalapril monotherapy in 7016 patients with NYHA class II-IV HF with a LVEF ≤ 35% and elevated plasma BNP levels (same criteria as in PARADIGM-HF). The main exclusion criteria were very similar to PARADIGM-HF, with more stringent requirements in renal function and serum potassium levels but a lower threshold of SBP. Patients were required to be treated with a beta-blocker (unless contraindicated) and could be treated with a MRA if felt to be indicated by the investigator. Patients were enrolled from March 13, 2009, to December 26, 2013 from 789 centers in 43 countries, and were followed up until July 31, 2015. The median follow-up was 36.6 months.

Both trials used a composite primary outcome of cardiovascular death or HF hospitalization. All patients provided written informed consent.

### Outcomes

In each trial, all deaths were adjudicated by the same committee using pre-specified criteria, in a blinded fashion. The same definitions for modes of death were used. SD was defined as death occurring unexpectedly in an otherwise stable patient, further classified as death witnessed or patient last seen alive < 1 h previously, and death in a patient last seen alive ≥ 1 h and < 24 h previously. PFD was defined as death occurring in the context of clinically worsening symptoms/signs of HF without evidence of another cause of death, including death as a complication of the implantation of a ventricular assist device, cardiac transplant or other surgery primarily for refractory HF, and death after referral to hospice specifically for progressive HF.

### Prediction variables

To identify predictors for each mode of death, a broad spectrum of baseline variables (*N* = 62) were separately assessed in PARADIGM-HF (Table 1). These variables included demographics, clinical variables, medical history, ECG parameters, and laboratory tests including NT-proBNP. In each trial, patient demographics and medical history were collected at baseline, physical examination, blood pressure, pulse and anthropometrical measurements were also performed, and this information was recorded in the electronic case report form (eCRF) by the investigators. A 12-lead ECG was performed at baseline and interpretation of the tracing was made by a qualified physician and documented on the ECG section of the eCRF. All laboratory tests were performed in a central laboratory, according to the pre-specified laboratory manual with details about specimen collections, shipment of samples and reporting of results, except potassium values and eGFR. These two tests were performed in a local laboratory and eGFR was calculated using the Modification of Diet in Renal Disease (MDRD) equation. A full set of baseline variables was collected in most patients, and patients with missing values were excluded in these analysis (< 2.5%). No difference was observed between the overall randomized patients and the cohort with all baseline variables available.

**Table 1:** Baseline patient characteristics in the derivation and validation trials

	PARADIGM-HF	ATMOSPHERE	
	*N* = 7156	*N* = 5968	
Age-years	63.7 ± 11.6	63.1 ± 12.1	0.01
Male sex—no. (%)	5492 (76.7)	4565 (76.5)	0.73
Race—no. (%)			< 0.001
White	4480 (62.6)	3659 (61.7)	
Black	344 (4.8)	95 (1.6)	
Asian	1480 (20.7)	1716 (28.9)	
Other	852 (11.9)	460 (7.8)	
Region—no. (%)			< 0.001
North America	275 (3.8)	81 (1.4)	
Latin America	1372 (19.2)	1077 (18.0)	
Western Europe	1423 (19.9)	1225 (20.5)	
Central Europe	2625 (36.7)	1737 (29.1)	
Asia or Pacific region	1461 (20.4)	1848 (31.0)	
Body mass index	28.0 ± 5.5	27.2 ± 5.3	< 0.001
Blood pressure-mmHg			
Systolic	122.0 ± 15.4	124.4 ± 18.2	< 0.001
Diastolic	74.2 ± 10.0	77.6 ± 11.0	< 0.001
Heart rate-beats/min	72.9 ± 12.1	72.4 ± 12.7	0.008
LVEF-%	29.9 ± 6.1	28.8 ± 5.5	< 0.001
NYHA class-no. (%)			< 0.001
I	347 (4.9)	164 (2.7)	
II	4988 (69.8)	4030 (67.5)	
III	1756 (24.6)	1718 (28.8)	
IV	54 (0.8)	56 (0.9)	
Ischemic etiology-no. (%)	4204 (58.7)	3232 (54.2)	< 0.001
HF duration-no. (%)			< 0.001
within 1 year	2391 (33.4)	2229 (37.4)	
> 1–5 years	2781 (38.9)	2197 (36.8)	
> 5 years	1984 (27.7)	1538 (25.8)	
Medical history-no. (%)			
Current smoking	1008 (14.1)	741 (12.4)	0.005
Previous HF hospitalization	4459 (62.3)	3490 (58.5)	< 0.001
Myocardial infarction	2919 (40.8)	2228 (37.3)	< 0.001
Angina	1944 (27.2)	1415 (23.7)	< 0.001
Stable angina	1547 (21.6)	1108 (18.6)	< 0.001
Unstable angina	768 (10.7)	600 (10.1)	0.21
CABG or PCI	1951 (27.3)	1475 (24.7)	0.001
Hypertension	5101 (71.3)	3725 (62.4)	< 0.001
Diabetes	2406 (33.6)	1629 (27.3)	< 0.001
Atrial fibrillation	2621 (36.6)	2002 (33.5)	< 0.001
Stroke	596 (8.3)	419 (7.0)	0.005
Cancer	320 (4.5)	186 (3.1)	< 0.001
Asthma	249 (3.5)	179 (3.0)	0.12
COPD	876 (12.2)	625 (10.5)	0.002
AAA	82 (1.1)	61 (1.0)	0.50
PAD	610 (8.5)	461 (7.7)	0.10
Medication-no. (%)			
Digoxin	2232 (31.2)	1940 (32.5)	0.11
Diuretics	5709 (79.8)	4713 (79.0)	0.25
ACE inhibitors	5527 (77.2)	5968 (100.0)	< 0.001
ARBs	1646 (23.0)	85 (1.4)	< 0.001
Beta-blockers	6610 (92.4)	5423 (90.9)	0.002
MRAs	3969 (55.5)	2109 (35.3)	< 0.001
Any antiplatelet agents	3988 (55.7)	3251 (54.5)	0.15
Aspirin	3653 (51.0)	3021 (50.6)	0.63
Anticoagulants	2173 (30.4)	1633 (27.4)	< 0.001
Statins	3796 (53.0)	2893 (48.5)	< 0.001
Pacemaker	513 (7.2)	358 (6.0)	0.007
CRT-P	136 (1.9)	107 (1.8)	0.65
12-lead ECG-no. (%)			
QRS duration -msec	114.3 ± 31.6	114.5 ± 31.6	0.78
Atrial fibrillation	1866 (26.1)	1434 (24.3)	0.02
Atrial flutter	66 (0.9)	52 (0.9)	0.80
Bundle branch block	1965 (27.5)	1659 (28.1)	0.43
Left bundle branch block	1440 (20.1)	1245 (21.1)	0.18
Right bundle branch block	552 (7.7)	441 (7.5)	0.59
Q wave	1247 (17.4)	1108 (18.8)	0.05
Left ventricular hypertrophy	1423 (19.9)	1189 (20.1)	0.73
Paced rhythm	421 (5.9)	296 (5.0)	0.03
Laboratory measurement			
eGFR < 60 ml/min/1.73 m^2^-no. (%)	2462 (34.4)	1467 (24.6)	< 0.001
eGFR-ml/min/1.73 m^2^	68.8 ± 20.3	75.1 ± 24.7	< 0.001
Creatinine-mg/dL	1.10 ± 0.29	1.02 ± 0.27	< 0.001
BUN-mmol/L	7.2 ± 2.9	7.2 ± 2.9	0.09
Albumin-g/L	42.8 ± 3.2	43.2 ± 3.6	< 0.001
Hemoglobin-g/L	139.4 ± 16.2	137.4 ± 16.6	< 0.001
Potassium-mmol/L	4.51 ± 0.48	4.46 ± 0.47	< 0.001
Sodium-mmol/L	141.5 ± 3.0	139.7 ± 3.3	< 0.001
Chloride-mmol/L	103.9 ± 3.4	103.8 ± 3.6	0.24
Calcium-mmol/L	2.32 ± 0.11	2.33 ± 0.12	0.06
Total cholesterol-mmol/L	4.59 ± 1.16	4.54 ± 1.20	0.005
HDL-C-mmol/L	1.24 ± 0.37	1.24 ± 0.38	0.50
LDL-C-mmol/L	2.58 ± 0.95	2.52 ± 1.00	< 0.001
Triglyceride-mmol/L	1.71 ± 1.18	1.75 ± 1.21	0.077
NT-proBNP-pg/ml^a^	1640 (888–3342)	1204 (630–2285)	< 0.001

^a^NT-proBNP measurements were available in 5408 patients in ATMOSPHERE

Plus-minus values are means ± standard deviations, NT-proBNP is presented as median with interquartile range. Other values are presented as number with percentage

*AAA* aortic artery aneurysm, *ACE* angiotensin-converting enzyme, *ARB* angiotensin receptor blocker, BUN blood urea nitrogen, *CABG* coronary artery bypass grafting, *COPD* chronic obstructive pulmonary disease, *CRT-P* cardiac resynchronization therapy with pacemaker, *ECG* electrocardiogram, *eGFR* estimated glomerular filtration rate, *HDL-C* high-density lipoprotein cholesterol, *HF* heart failure, *LDL-C* low-density lipoprotein cholesterol, *LVEF* left ventricular ejection fraction, *MRA* mineralocorticoid receptor antagonist, *NT-proBNP* N-terminal pro-B-type natriuretic peptide, *NYHA* New York Heart Association, *PAD* peripheral artery disease, *PCI* percutaneous coronary intervention

### Statistical analysis

The baseline characteristics by cohort were compared using Student’s *t* test or Mann–Whitney *U* test as appropriate for continuous variables, and Chi-square test for categorical variables. For each mode of death, the event rate was calculated per 100 patient-years, and the cumulative incidences over time were plotted and compared by cohort using the Pepe–Mori test which counted death from other causes as a competing risk.

A univariable Fine and Gray sub-distribution hazards model was first performed to assess the influence of each prediction variable on the cumulative incidence of each mode of death [17]. For each continuous variable, linearity was examined using the restricted cubic spline method. If the response appeared nonlinear, certain cut-off values or transformation were applied according to the spline curves and clinical relevance. For categorical variables, appropriate dummy variables were used. The validity of the proportional sub-distribution hazards assumption was examined using time varying terms. For each variable, the statistical strength for predicting each mode of death was quantified by *Χ*^2^ values with one degree of freedom.

For each outcome, we used a multivariable Fine–Gray model with backward stepwise selection based on Akaike information criterion (i.e., equivalent to *p* = 0.157), starting with a full model including all candidate variables. The predictor selection process was repeated in 200 bootstrap samples, each was sampled with replacement from the original PARADIGM-HF dataset with the same sample size as the original. To minimize the chance of inclusion of weak and uninformative predictors which might lead to model overfitting and optimism, we included variables in the final model that were retained in > 50% of all bootstrap datasets and were statistically significant. Since LVEF is an established prognostic factor for pump failure death, we included it in the final model regardless of the abovementioned inclusion criteria. For each mode of death, the final model was refitted into 200 bootstrap samples to get the average predictor coefficients. These averaged coefficients were used to calculate the individual risk score which is the sum of the products of each predictor value and its corresponding coefficient. Predicted cumulative incidences over time by quartile of risk scores were plotted against the observed Aalen-Johansen estimators to assess model performance [18]. Model calibration was examined by comparing observed-predicted pairs of curves in each quartile over time. Model discrimination was examined by visually assessing the spread of each set of curves (the wider the better) and by calculating Harrell’s C and C-index at 1-, 2-, and 3-year adjusting for right censoring [19].

To correct for optimism, internal validation was undertaken by bootstrapping approach. In detail, the C statistic of the derived model was determined in each bootstrap sample from which it was generated, and also in the original dataset, and the difference between these two C statistics was calculated and then averaged over 200 samples to give an estimate of the optimism. The optimism corrected estimate of the C statistic was then calculated as the naïve C statistic minus the estimated optimism. External validation was performed in the ATMOSPHERE cohort by fitting a univariable Fine–Gray regression on risk score which was the sum of average coefficients of predictors for each model from PARADIGM-HF multiplied by its corresponding predictor values in ATMOSPHERE. Model performance in validation was assessed using the same approach mentioned above.

To determine whether the prediction variables had a different effect on each outcome, all predictors from both models were fitted into cause-specific Cox regression models using the Lunn–McNeil method [20].

To validate the SHFM in contemporary cohorts and to compare our models with the SHFM, a SHFM score was calculated for each patient in PARADIGM-HF and ATMOSPHERE and the ability of the SHFM to discriminate between SD and PFD was assessed. We also validated the SPRM in both cohorts using logistic regression analysis and assessed its discrimination using Receiver Operating Characteristic Area Under the Curve (ROC AUC), an equivalent to Harrell’s C.

A two-tailed *p* < 0.05 was considered significant. The cumulative incidence function and C-index were achieved using the ‘cmprsk’ and ‘pec’ packages in R project (version 3.2.3). Other analyses were performed using STATA software (version 14.0 SE).

## Results

### Patient characteristics in the derivation cohort (PARADIGM-HF)

The derivation cohort included 7156 patients from PARADIGM-HF after excluding 1243 patients with an ICD or CRT-D. As can be seen in Table 1, there was a predominance of males (77%) with a mean age of 63.7 years. The mean LVEF was 29.9%, the vast majority of patients were in NYHA class II–III (mainly in class II) and most had an ischemic etiology (58.7%). There was a high rate of evidence-based treatment with 92.4% having a beta-blocker and 55.5% receiving a MRA.

### Derivation of mode-specific death models

In PARADIGM-HF, there were 1344 death events including 525 SD and 261 PFD over a median follow-up of 27 months. The annual rate was 3.4 (95% CI 3.1–3.7) per 100 patient-years for SD and 1.7 (95% CI 1.5–1.9) per 100 patient-years for PFD, respectively.

Table 2 shows the 25 most powerful predictors for SD from the univariate analysis, and 10 of them were independent predictors for SD in the multivariable model: male sex, Asian or Black race, NYHA class III/IV vs. I/II, prior CABG or PCI, history of myocardial infarction, cancer history, treatment with LCZ696 compared with enalapril, left ventricular hypertrophy (LVH) on ECG, QRS duration (90–120 ms), and plasma NT-proBNP (log-transformed).

**Table 2:** Univariate and multivariable predictors for sudden death in PARADIGM-HF

	Univariate analysis^a^			Multivariable model			
	sHR (95% CI)	*p* value	*X*^2^ score	sHR (95% CI)	Coefficient†	*p* value	*X*^2^ score
log NT-proBNP -pg/mL (from 6), per 1 log	1.50 (1.38–1.62)	< 0.001	96.2	1.41 (1.31–1.53)	0.348	< 0.001	71.2
Asian or Black race	1.69 (1.41–2.02)	< 0.001	32.6	1.82 (1.51–2.19)	0.581	< 0.001	39.1
Asian race	1.71 (1.40–2.08)	< 0.001	28.5				
LVEF 25–35%, per 1%	0.95 (0.93–0.97)	< 0.001	19.5				
NYHA class III/IV vs. I/II	1.48 (1.23–1.77)	< 0.001	17.6	1.40 (1.16–1.70)	0.338	0.001	11.9
Body mass index 17–28 kg/m^2^, per 1 kg/m^2^	0.94 (0.92–0.97)	< 0.001	16.1				
QRS duration 90–120 ms, per 5 ms	1.07 (1.03–1.10)	< 0.001	14.0	1.07 (1.03–1.11)	0.065	< 0.001	14.7
Triglycerides up to 2.3 mmol/L, per 1 mol/L	0.74 (0.62–0.87)	< 0.001	14.0				
MI history	1.35 (1.14–1.61)	0.001	12.0	1.79 (1.47–2.17)	0.582	< 0.001	33.5
CABG or PCI	0.70 (0.56–0.86)	0.001	11.5	0.61 (0.48–0.77)	− 0.502	< 0.001	16.8
Male sex	1.47 (1.18–1.85)	0.001	11.2	1.43 (1.14–1.80)	0.361	0.002	9.2
Cancer history	0.31 (0.16–0.63)	0.001	10.6	0.37 (0.19–0.76)	− 1.098	0.006	7.5
Left ventricular hypertrophy on ECG	1.36 (1.12–1.66)	0.002	9.5	1.27 (1.04–1.56)	0.233	0.021	5.3
Ischemic etiology	1.30 (1.09–1.56)	0.004	8.4				
Albumin 30–45 g/L, per 1 g/L	0.96 (0.92–0.99)	0.004	8.4				
Hemoglobin A1C 5.7–12.0%, per 1%	1.09 (1.03–1.16)	0.004	8.3				
Systolic BP up to 130 mmHg, per 5 mmHg	0.95 (0.91–0.98)	0.005	8.0				
Black race	1.69 (1.17–2.44)	0.005	7.9				
Serum potassium up to 4.0 mmol/L, per 0.1 mmol/L	0.92 (0.82–0.98)	0.006	7.4				
Bundle branch block on ECG	1.28 (1.06–1.53)	0.009	6.9				
Digoxin use	1.23 (1.03–1.47)	0.024	5.1				
Beta-blocker use	0.73 (0.55–0.97)	0.031	4.7				
LCZ696 vs. enalapril	0.83 (0.70–0.98)	0.032	4.6	0.83 (0.70–0.99)	− 0.175	0.038	4.3
Asthma history	0.51 (0.28–0.95)	0.033	4.5				
Atrial fibrillation history	0.82 (0.68–0.98)	0.033	4.5				

*BP* blood pressure, *CABG* coronary artery bypass grafting, *CI* confidence interval, *LVEF* left ventricular ejection fraction, *NT-proBNP* N-terminal pro-B-type natriuretic peptide, *NYHA* New York Heart Association, *PCI* percutaneous coronary intervention, *sHR* sub-distribution hazard ratio

^a^The 25 strongest variables from the univariate analysis are presented

^†^Each coefficient of the predictors in the final model was the average of the predictor coefficients from 200 bootstrap samples

The 25 strongest predictors for PFD from the univariate analysis are displayed in Table 3, 11 of which were included in the multivariable model: SBP (up to 130 mmHg), NYHA class III/IV vs. I/II, LVEF (up to 40%), ischemic etiology, a diagnosis of HF for > 5 years, HF duration > 1 and ≤ 5 years, bundle branch block (BBB) on ECG, serum albumin concentration (30–45 g/L), creatinine (1.0–2.5 mg/dL) and chloride (90–106 mmol/L), and plasma NT-proBNP (log-transformed).

**Table 3:** Univariate and multivariable predictors for pump failure death in PARADIGM-HF

	Univariate analysis^a^			Multivariable model			
	sHR (95% CI)	*p* value	*X*^2^ score	sHR (95% CI)	Coefficient†	*p* value	*X*^2^ score
Log NT-proBNP-pg/ml (from 6), per 1 log	2.00 (1.78–2.24)	< 0.001	142.3	1.61 (1.41–1.84)	0.473	< 0.001	49.4
Albumin 30–45 g/L, per 1 g/L decrease	1.16 (1.12–1.20)	< 0.001	62.7	1.10 (1.06–1.15)	0.102	< 0.001	26.5
BUN 6–16 mmol/L, per 1 mmol/L	1.17 (1.12–1.22)	< 0.001	54.6				
Creatinine 1.0–2.5 mg/dL, per 0.1 mg/dL	1.15 (1.10–1.19)	< 0.001	48.9	1.08 (1.03–1.13)	0.076	0.001	11.4
eGFR up to 60 ml/min/1.73 m^2^, per 1 ml/min/1.73 m^2^	0.96 (0.94–0.97)	< 0.001	42.1				
Serum chloride 90–106 mmol/L, per 1 mmol/L decrease	1.12 (1.08–1.16)	< 0.001	38.3	1.09 (1.06–1.13)	0.091	< 0.001	24.3
HF duration > 5 years vs. ≤ 1 year	2.71 (1.94–3.80)	< 0.001	34.0	2.58 (1.81–3.68)	0.961	< 0.001	27.5
LVEF up to 40%, per 1% decrease	1.05 (1.03–1.07)	< 0.001	30.0	1.02 (1.00–1.04)	0.020	0.032	4.6
Hemoglobin 90–140 g/L, per 1 g/L	0.97 (0.96–0.98)	< 0.001	26.9				
Systolic BP up to 130 mmHg, per 5 mmHg decrease	1.14 (1.08–1.21)	< 0.001	22.8	1.10 (1.04–1.17)	0.096	0.001	12.1
QRS duration 90–220 ms, per 5 ms	1.05 (1.03–1.06)	< 0.001	22.8				
Pacemaker implanted	2.29 (1.62–3.23)	< 0.001	22.0				
Diastolic BP -mmHg, per 1 mmHg	0.97 (0.96–0.98)	< 0.001	19.7				
Bundle branch block on ECG	1.72 (1.34–2.21)	< 0.001	18.5	1.45 (1.12–1.87)	0.384	0.005	8.1
Cardiac resynchronization therapy	2.96 (1.68–5.22)	< 0.001	14.1				
Age 70–96 years, per 1 year	1.05 (1.02–1.08)	< 0.001	14.1				
Serum sodium -mmol/L, per 1 mmol/L	0.93 (0.89–0.97)	< 0.001	12.8				
Digoxin use	1.56 (1.22–1.99)	< 0.001	12.4				
Diuretics use	1.97 (1.35–2.88)	< 0.001	12.3				
NYHA class III/IV vs. I/II	1.57 (1.22–2.03)	0.001	12.1	1.42 (1.08–1.86)	0.354	0.011	6.5
Ischemic etiology	0.66 (0.52–0.84)	0.001	11.4	0.70 (0.54–0.90)	-0.372	0.005	7.7
Hemoglobin A1C -%, per 1%	1.11 (1.04–1.18)	0.001	10.4				
Right bundle branch block on ECG	1.81 (1.26–2.59)	0.001	10.3				
HF duration > 1–5 years vs. ≤ 1 year	1.73 (1.23–2.43)	0.002	9.9	1.73 (1.21–2.47)	0.549	0.003	9.1
Heart rate above 70 beats/min, per 1 beat/min	1.02 (1.01–1.03)	0.002	9.7				

*BP* blood pressure, *BUN* blood urea nitrogen, *CI* confidence interval, *eGFR* estimated glomerular filtration rate, *HF* heart failure, *LVEF* left ventricular ejection fraction, *NT-proBNP* N-terminal pro-B-type natriuretic peptide, *NYHA* New York Heart Association, *sHR* sub-distribution hazard ratio

^a^The 25 strongest variables from the univariate analysis are presented

^†^Each coefficient of the predictors in the final model was the average of the predictor coefficients from 200 bootstrap samples

### Performance of the models

**Fig. 1:**
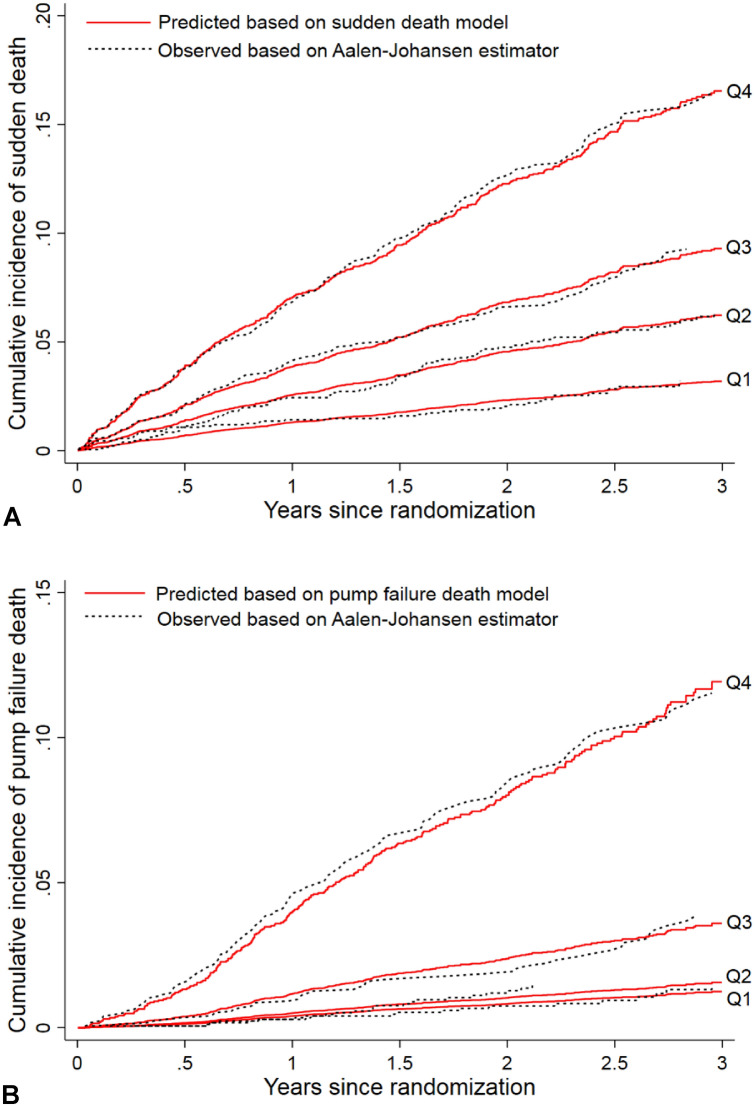
Observed vs. predicted cumulative incidence curves for sudden death and pump failure death by quartile of risk scores based on the corresponding models in PARADIGM-HF. **A** Sudden death model; **B** pump failure death model. Red solid lines are predicted cumulative incidence curves based the corresponding models, and black dotted lines are the observed cumulative incidence curves based on Aalen–Johansen estimators. *Q1* the quartile 1, *Q2* the quartile 2, *Q3* quartile 3, *Q4* quartile 4

The SD model showed good discrimination, with Harrell’s C of 0.68 (95% CI 0.66–0.71) and C-index of 0.67, 0.68 and 0.67 at 1, 2 and 3 years, respectively. With boot-strapping interval validation, the Harrell’s C corrected for optimism was 0.67. The curves for observed Aalen–Johansen estimators and predicted cumulative incidences were almost identical over time in each quartile of risk score based on the model, indicating good calibration (Fig. 1a). Both sets of quartile risk curves are well separated, confirming the discrimination suggested by the C statistics.

The PFD model also calibrated well: the curves for the predicted cumulative incidence agreed well with the corresponding observed curves for each quartile of risk score (Fig. 1b). Although the PFD model was less able to distinguish between the lowest two risk quartiles, it identified the highest and second-highest quartiles, which had over 10 times and 3 times the risk, respectively, of the lowest quartile at 3 years (Fig. 1b). The excellent discrimination was confirmed by high Harrell’s C values of 0.79 (95% CI 0.76–0.82) and C-index of 0.82, 0.79 and 0.77 over 1, 2 and 3 years, separately. With boot-strapping interval validation, the Harrell’s C corrected for optimism was 0.78.

Some violation of proportional sub-distribution hazards assumption was observed with albumin (*p* = 0.02) for the PFD model. However, when presented graphically, the curves for the cumulative incidences by tertile of each predictor did not cross over time, indicating the breach was acceptable (Online Fig. A1).

### External validation of the current models in ATMOSPHERE

External validation was performed in 5968 patients in ATMOSPHERE after excluding 1048 patients with an ICD or CRT-D. The baseline characteristics of these patients were similar, for the most part, to PARADIGM-HF. However, some differences were observed. In ATMOSPHERE, there was a higher proportion of Asian patients, but a lower proportion of patients with a history of hypertension, diabetes or renal dysfunction. Fewer patients in ATMOSPHERE had received a MRA. The median plasma NT-proBNP level was lower in ATMOSPHERE. Patient characteristics by cohort are summarized in Table 1.

During a median 37.7 months of follow-up, 1644 death events occurred in ATMOSPHERE including 607 SD and 305 PFD, with the corresponding annual rates very similar to PARADIGM-HF. The cohort-specific cumulative incidences for SD were nearly identical, and this was also the case for PFD (Online Fig. A2).

**Fig. 2:**
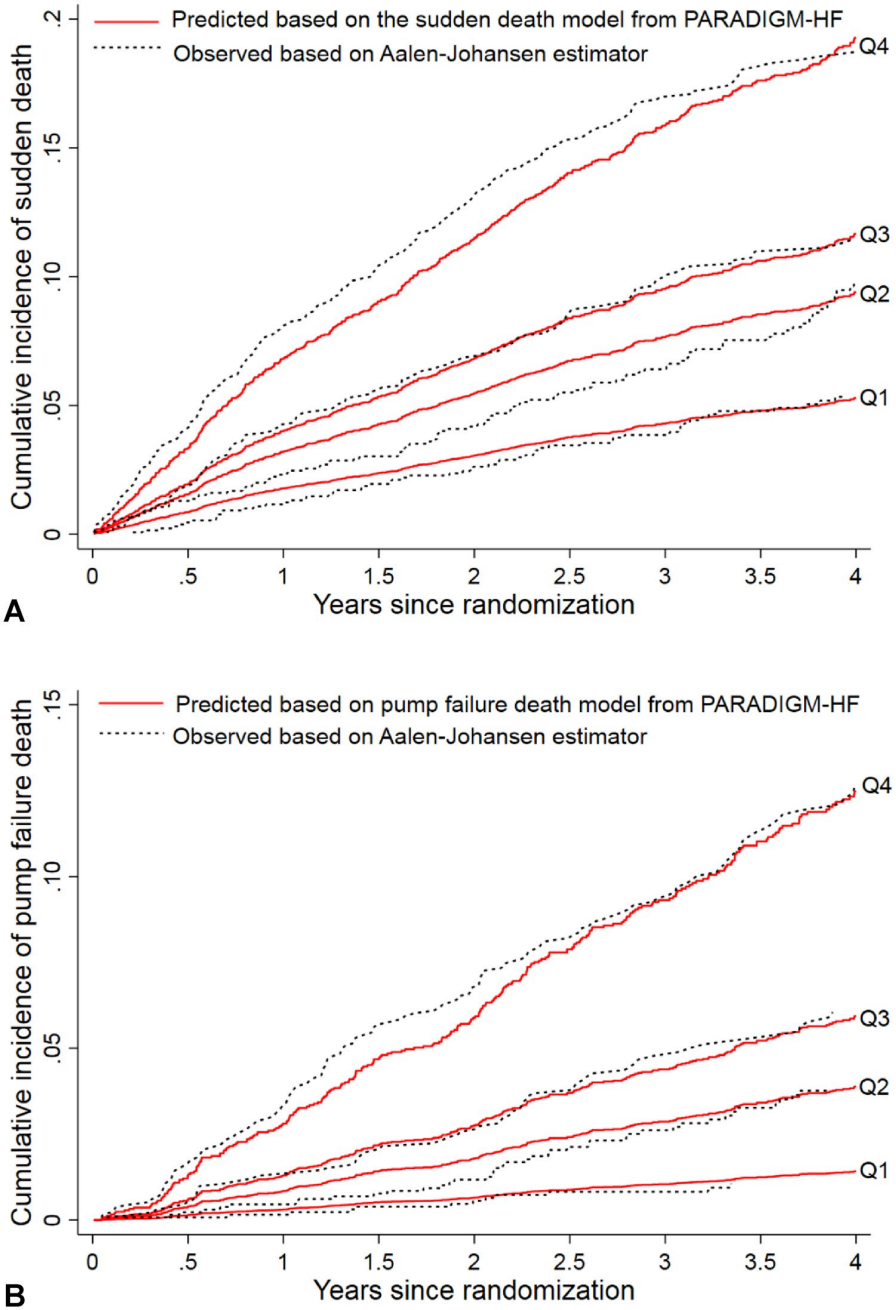
Observed vs. predicted cumulative incidence curves for sudden death and pump failure death by quartile of risk scores based on the corresponding models from PARADIGM-HF in ATMOSPHERE. **A** Sudden death model; **B** pump failure death model. Red solid lines are predicted cumulative incidence curves based the corresponding models, and black dotted lines are the observed cumulative incidence curves based on Aalen–Johansen estimators. *Q1* the quartile 1, *Q2* the quartile 2, *Q3* quartile 3, *Q4* quartile 4

For the SD model, discrimination was largely stable in the validation cohort with a Harrell’s C of 0.66 (95% CI 0.64–0.69) and C-index of 0.71, 0.68 and 0.67 at 1, 2 and 3 years, respectively. Although the highest quartile under-predicted the cumulative incidence while the second-lowest quartile over-estimated the rate over time, the predicted and observed cumulative incidences were broadly similar in the rest quartiles (Fig. 2a).

For the PFD model, discrimination was slightly decreased but remained robust in the validation cohort with a Harrell’s C of 0.75 (95% CI 0.72–0.78) and C-index of 0.78, 0.76 and 0.73 at 1, 2 and 3 years, respectively. Calibration was reasonable except in the highest risk subgroup, where an underestimation was observed in the early period of follow-up (Fig. 2b).

### Predicting “individual risk”

**Fig. 3:**
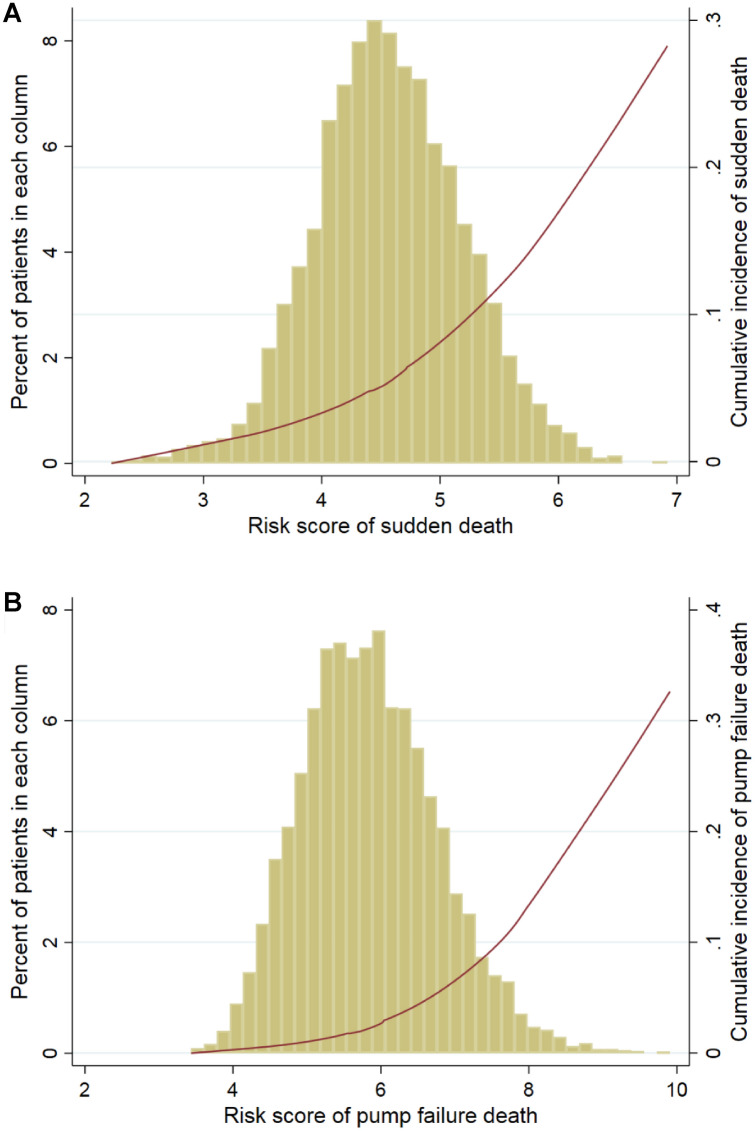
Distributions of risk scores for sudden death and pump failure death and its relation to the corresponding cumulative incidence within 3 years in PARADIGM-HF. **A** The risk score and the corresponding cumulative incidence based on sudden death model; **B** The risk score and the corresponding cumulative incidence based on pump failure death model. The columns are the histogram of the risk score for mode-specific death, the left axis shows the percent of patients in each column. The red line is the cumulative incidences of mode-specific death for the corresponding risk scores

The probability that out of a (hypothetical) population of 100 patients with the same characteristics as an observed patient, *x*% will have the event is often loosely described as an “individual risk”. Such a risk for SD and PFD can be calculated by adding up the products of their predictor values and the coefficients from the multivariable models presented in Tables 2 and 3, respectively. For the obtained risk scores, the corresponding cumulative incidences for SD and PFD within 3 years can be estimated using the curves displayed in Fig. 3a, b which shows the distribution of the risk score for each mode of death and its relationship with the corresponding predicted cumulative incidence within 3 years, respectively (Examples are given in Online Supplement).

### Relationships between specific prognostic variables and SD, compared with PFD

NYHA class and NT-proBNP were included in both models. More advanced NYHA class and higher NT-proBNP were associated with higher risks of both modes of death, with both associations stronger for PFD than SD, although the differences did not reach significance (Table 4). Ischemic etiology had an opposite association with mode-specific death, i.e. it was associated with a higher risk of SD but a lower risk of PFD.

**Table 4:** Differences in associations between prognostic variables and sudden death, compared with pump failure death, in the multivariable analysis

	Sudden death		Pump failure death		*p* value for difference
	HR (95% CI)	*p* value	HR (95% CI)	*p* value	
Male sex	1.42 (1.12–1.79)	0.004	1.12 (0.81–1.54)	0.492	0.246
Asian or Black race	1.72 (1.41–2.10)	< 0.001	1.17 (0.87–1.57)	0.311	0.034
MI history	1.53 (1.20–1.95)	< 0.001	1.38 (0.93–2.03)	0.109	0.645
Left ventricular hypertrophy on ECG	1.30 (1.06–1.60)	0.012	0.90 (0.65–1.25)	0.543	0.064
CABG or PCI	0.57 (0.45–0.73)	< 0.001	0.75 (0.52–1.07)	0.113	0.229
Cancer history	0.39 (0.19–0.78)	0.008	1.05 (0.60–1.85)	0.87	0.03
LCZ696 vs. enalapril	0.82 (0.69–0.98)	0.029	0.86 (0.67–1.11)	0.248	0.754
QRS duration 90–120 ms, per 5 ms	1.06 (1.02–1.11)	0.003	1.06 (0.99–1.12)	0.062	0.894
NYHA class III/IV vs. I/II	1.38 (1.13–1.68)	0.001	1.52 (1.16–1.99)	0.002	0.565
Log NT-proBNP -pg/ml (from 6), per 1 log	1.45 (1.32–1.59)	< 0.001	1.65 (1.45–1.88)	< 0.001	0.106
Ischemic etiology	1.28 (1.00–1.64)	0.047	0.63 (0.44–0.93)	0.018	0.002
Systolic BP up to 130 mmHg, per 5 mmHg	0.96 (0.92–1.00)	0.04	0.90 (0.85–0.95)	< 0.001	0.067
LVEF up to 40%, per 1%	0.99 (0.98–1.01)	0.334	0.98 (0.96–1.00)	0.055	0.335
Bundle branch block on ECG	1.09 (0.88–1.35)	0.448	1.27 (0.94–1.70)	0.114	0.408
HF duration > 1–5 years vs. ≤ 1 year	1.14 (0.93–1.41)	0.216	1.73 (1.21–2.46)	0.002	0.049
HF duration > 5 years vs. ≤ 1 year	1.16 (0.91–1.47)	0.237	2.51 (1.75–3.59)	< 0.001	< 0.001
Creatinine 1.0–2.5 mg/dl, per 0.1 mg/dl	0.99 (0.96–1.03)	0.726	1.07 (1.03–1.12)	0.002	0.01
Chloride 90–106 mmol/L, per 1 mmol/L	0.97 (0.94–1.00)	0.046	0.90 (0.87–0.94)	< 0.001	0.003
Albumin 30–45 g/L, per 1 g/L	0.98 (0.95–1.01)	0.141	0.89 (0.86–0.93)	< 0.001	< 0.001

*BP* blood pressure, *CABG* coronary artery bypass graft, *CI* confidence interval, *HF* heart failure, *HR* hazard ratio, *LVEF* left ventricular ejection fraction, *NT-proBNP* N-terminal pro-B-type natriuretic peptide, *NYHA* New York Heart Association, *PCI* percutaneous coronary intervention

Hazard ratio is for the presence versus absence of dichotomous variables, and per increase in continuous variables as specified in the table with all covariates showed in the model simultaneously

Male sex, Asian or Black race, a history of MI and LVH on ECG were associated with a higher risk of SD, whereas history of cancer, prior CABG or PCI and treatment with LCZ696 (compared with enalapril) were associated with a lower risk of SD, but none of these variables were predictive for PFD (Table 4). On the contrary, longer HF duration, a higher level of serum creatinine, and lower serum albumin or chloride were associated with a higher risk of PFD but not of SD (*p* for inequality all < 0.05).

### Validation of the SHFM and SPRM in PARADIGM-HF and ATMOSPHERE

The SHFM is reported to show good discrimination for SD and PFD with 1-year ROC AUC of 0.68 (95% CI 0.65–0.70) and 0.85 (95% CI 0.83–0.87), respectively [12]. However, when validated in our more contemporary cohorts, its discrimination declined for SD, with C statistics of 0.57 (0.53–0.60) at 1 year and 0.58 (0.55–0.60) at 3 years in PARADIGM-HF and 0.62 (0.58–0.66) at 1 year and 0.63 (0.61–0.66) at 3 years in ATMOSPHERE. A marked decrease in discrimination from that reported was also observed for PFD, with 1- and 3-year C statistics of 0.72 (0.67–0.77) and 0.69 (0.66–0.72) in PARADIGM-HF and 0.71 (0.64–0.77) and 0.65 (0.61–0.69) in ATMOSPHERE.

The SPRM showed poor discrimination for SD in PARADIGM-HF (ROC AUC 0.57) and ATMOSPHERE (ROC AUC 0.54). Using a 25% of 1-year mortality rate and a threshold proportion of mortality due to SD of 42% (used by the SPRM authors to identify patients most likely to benefit from an ICD), this model allocated most patients to the upper left quadrant, i.e. those who had a low mortality, primarily due to SD, and an indication for an ICD (Online Fig. A3).

## Discussion

We developed and validated separate prognostic models for SD and PFD in patients with HFrEF enrolled in PARADIGM-HF and ATMOSPHERE, the two largest and most contemporary trials in HF, using a competing risk analysis approach. Both models showed good discrimination and calibration and remained robust in the external validation.

The potential value of estimating the risk for mode-specific death, and in particular SD, in individual HF patients, has recently been reinforced by the results of the Danish Study to Assess the Efficacy of ICDs in Patients with Non-ischemic Systolic Heart Failure on Mortality (DANISH) [21]. In DANISH, ICD treatment did not reduce overall mortality in patients at low risk of SD as a result of excellent contemporary therapy [21]. Older individuals, with more co-morbidity, were least likely to benefit, probably because they had a higher competing risk of PFD, and non-cardiovascular causes of death, both of which would not be reduced by an ICD [21, 22]. This trial raises the question of whether ICD implantation might be better targeted to individuals at highest risk of SD [21, 23]. As the contemporary risk of sudden death declines, concern has been expressed as to whether the benefits of ICDs outweigh the risks of these devices when applied in a relatively un-targeted way [14, 24]. For example, in a recent nationwide analysis of complications after primary prevention ICD implantation in ambulatory patients in the USA, the device-related mortality rate was reported to be 0.73% at 30 days, with a total serious complication rate of 8.4% [25]; similar data have been reported from other countries [26].

At least one recent guideline has suggested that validated risk calculators/risk assessment tools may “aid in the estimation of each patient’s benefit/risk of an ICD implantation” [3]. Several models for predicting modes of death in HF already exist. However, because they all have limitations, none has gained widespread acceptance in current clinical practice. Older models were developed before the broad utilization of contemporary evidence-based medications, e.g. beta-blockers and MRAs [10, 11]. More recently, separate risk scores for SD and PFD were reported among HF patients with unspecified left ventricular function in the MUSIC study [8]. Although the models offered excellent discrimination, with c-indices of 0.77 for SD and 0.80 for PFD, they were based on a small number of events (90 SD and 123 PFD) and few candidate predictors. Models for SD and PFD were also developed in CORONA but only patients with an ischemic etiology were included in that trial [9]. Moreover, the CORONA model did not include routinely collected variables, such as serum chloride and albumin. Likewise, the HF-ACTION investigators only assessed the additional mode-specific death information gained from adding biomarkers, i.e. NT-proBNP, galectin-3 and soluble ST2, to a clinical model developed previously for all-cause death [6, 27]. No prediction model or risk score was provided and over 46% of patients in HF-ACTION had an ICD in situ. Importantly, none of the models mentioned accounted for competing risks from other deaths or were validated in an independent population.

Although the SHFM reported good discrimination for predicting SD and, particularly, PFD, comparable to the models developed in this study [4, 12], when it was applied to PARADIGM-HF and ATMOSPHERE, there was a substantial decline in its ability to discriminate, indicating a significant loss of power to predict mode-specific death in a contemporary population receiving evidence-based medications according to current guidelines. Moreover, the predictive variables in SHFM more reflect overall survival, and lack specificity for each mode of death.

The SPRM was recently developed to predict the proportion of mortality due to SD rather than the absolute risk of SD [13, 28, 29]. Using the predicted annual total mortality rate derived from SHFM, the authors attempted to identify a subset of patients who would benefit most from ICD, based on having a high risk of SD but a low risk of dying from other causes. However, when this bi-modal system was applied to each of our more contemporary cohorts, it yielded poor discrimination, assigning most patients to ICD implantation. This poor performance in identifying potential candidates for ICD implantation may reflect a difference in the underlying risk across the cohorts, particularly the proportion of sudden to overall death in the validation cohorts (< 40%) and the derivation cohort (48%) [13, 15, 16]. Thus, the intercept from the original SPRM may not be transportable, and its direct application may lead to the predicted proportional risk being systematically higher in validation cohorts. However, in patients with non-ischemic heart failure randomized in the DANISH trial, ICD use was associated with a lower mortality among patients with both a SPRM and SHFM score above the median, i.e. these scores may be better at predicting response to ICD therapy than in identifying patients for implantation, at least in patients with a particular etiology (30).

The models developed in our study have some unique features and, as a result, strengths. They are based on a large contemporary population with a substantial number of patients receiving modern evidence-based therapies. Additionally, we examined a broad spectrum of candidate variables which are currently assessed in clinical practice, many of which have been reported to predict SD [13, 30–32], including demographics, physical examination, medical history, treatment, ECG, routine biochemical tests and new biomarkers (such as NT-proBNP). Also, death from other causes was treated as a competing risk rather than non-informative censoring, which diminishes bias related to each individual mode of death [33]. More importantly, our models were validated with robust results in an independent cohort. Given the geographically and ethnically diverse cohorts included, our models should be generalizable to a broad range of contemporary patients with HFrEF.

Of special interest are the similarities and differences between predictive variables for each mode of death. Advanced NYHA class, lower SBP and elevated NT-proBNP levels were predictive of both modes of death (and there was a strong trend for ECG QRS duration). Three variables showed a similar directional association with each mode of death but a stronger relationship with one mode over the other: longer duration of HF (PFD), serum albumin (PFD) and chloride (PFD), all indicators of more advanced heart failure. Four variables had directionally opposite relationships with each mode of death. Ischemic etiology was independently associated with a higher risk of SD but with a lower risk of PFD (a similar trend was seen for ECG left ventricular hypertrophy). Higher creatinine was associated with a higher risk of PFD and history of cancer was associated with a lower risk of SD.

Although LVEF is a well-known predictor for SD, and is recommended as the key criterion for selecting ICD recipients [2, 31], we found it was neither independently associated with SD nor differentiated between SD and PFD in the present models. This may reflect the relatively narrow range of LVEF among patients enrolled in PARADIGM-HF and, possibly, the inclusion of NT-proBNP in our models. NT-proBNP level was somewhat more strongly associated with the risk of SD than PFD, although the difference was not statistically significant. This hypothesis might also explain the under-estimation of the rate of SD in the highest risk quartile in the validation model as NT-proBNP concentration was slightly lower in ATMOSPHERE than in PARADIGM-HF.

There are several limitations to the present analysis. First, our models were built and validated in clinical trials rather than in “real-world” cohorts, that is, patients in trials tend to be healthier, have less co-morbidity and be more likely to receive evidence-based therapies. However, it is in patients similar to those in the present study in which ICDs are most clearly indicated. Second, our SD model was less discriminative than the PFD model, as previously reported for other models of SD [8, 12]. Some variables reported predictive of SD and PFD were not measured in PARADIGM-HF including echocardiographic parameters, ambulatory ECG findings [8], and other biomarkers [27]. Third, ICDs can change the mode of death in a given patient. Although patients with an ICD at baseline were excluded, we cannot rule out the potential confounding effect of ICDs implanted after randomization, although there were few such cases (2.7%). Furthermore, even if mode-specific death is appropriately classified and predicted by the models with reasonable accuracy, this might not translate into prediction of the response to treatment. This is particularly because not all sudden deaths are electrical and preventable by an ICD (some may be due to other types of cardiovascular events). Lastly, we did not account for heart transplantation and ventricular assist device implantation during follow-up, although there were very few such procedures.

## Conclusion

Using the two largest and most contemporary trials in patients with HFrEF, we developed and validated robust models which separately predict SD and PFD. These models may help clinicians and patients when considering therapies targeted at these modes of death and in the selection of patients for specific interventions in future trials.

## Supplementary Information

Below is the link to the electronic supplementary material.

Supplementary file1 (DOCX 787 kb)

## Data Availability

The data used in this study are not available to others. Novartis is committed to sharing with qualified external researchers, access to patient-level data and supporting clinical documents from eligible studies. These requests are reviewed and approved by an independent review panel on the basis of scientific merit. This trial data availability is according to the criteria and process described on www.clinicalstudydatarequest.com.

## Code availability

STATA
