# Supplementary material for: Development and external validation of prognostic models to predict sudden and pump-failure death in patients with HFrEF from PARADIGM-HF and ATMOSPHERE
Source: Clin Res Cardiol. 2021 Jun 8;110(8):1334–49. doi: 10.1007/s00392-021-01888-x (PMC8318968; doi:10.1007/s00392-021-01888-x)
Supplement: Supplementary file 1 — Supplementary file1 (DOCX 787 kb) [file 392_2021_1888_MOESM1_ESM.docx]

ONLINE SUPPLEMENTAL MATERIAL

**Online Figures**

**Online Fig. A1** Cumulative incidence curve of pump failure death by tertile of serum albumin level in PARADIGM-HF

**Online Fig. A2** Cohort-specific cumulative incidence curves for sudden death and for pump failure death

**Online Fig. A3** Validation of Seattle Proportional Risk model in PARADIGM-HF and ATMOSPHERE

**Online Supplement** Examples of risk prediction for mode-specific death using the models presented in Table 2 and 3.

**Online Fig. A1** Cumulative incidence curve of pump failure death by tertile of serum albumin level in PARADIGM-HF

**
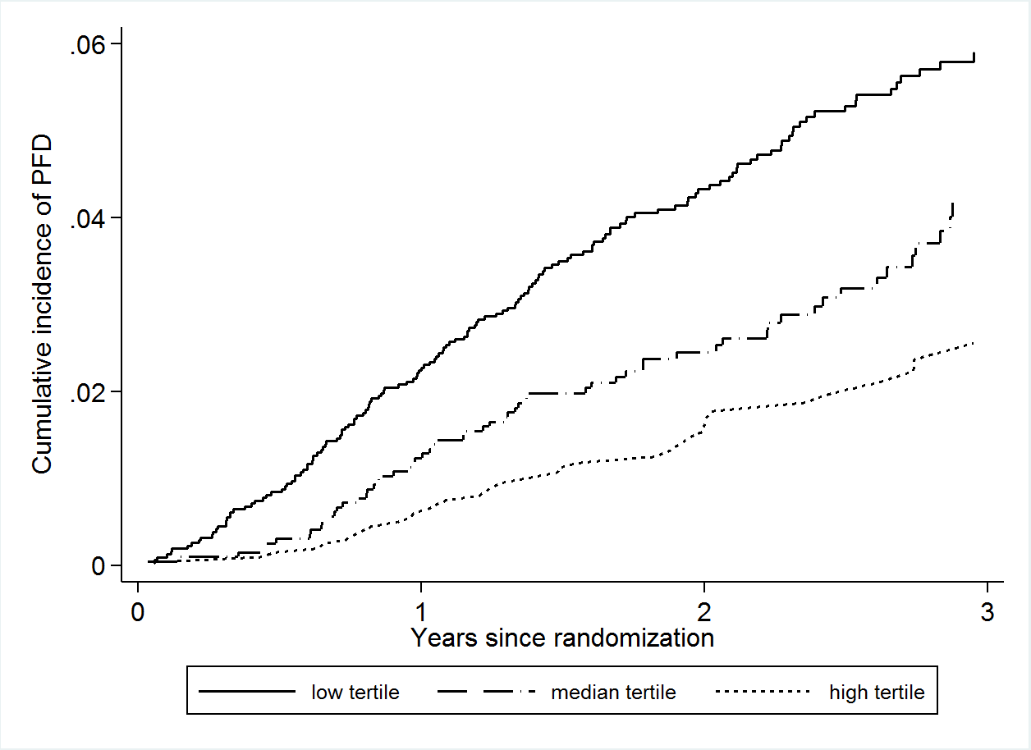
**

**Online Fig. A2** Cohort-specific cumulative incidence curves for sudden death and for pump failure death


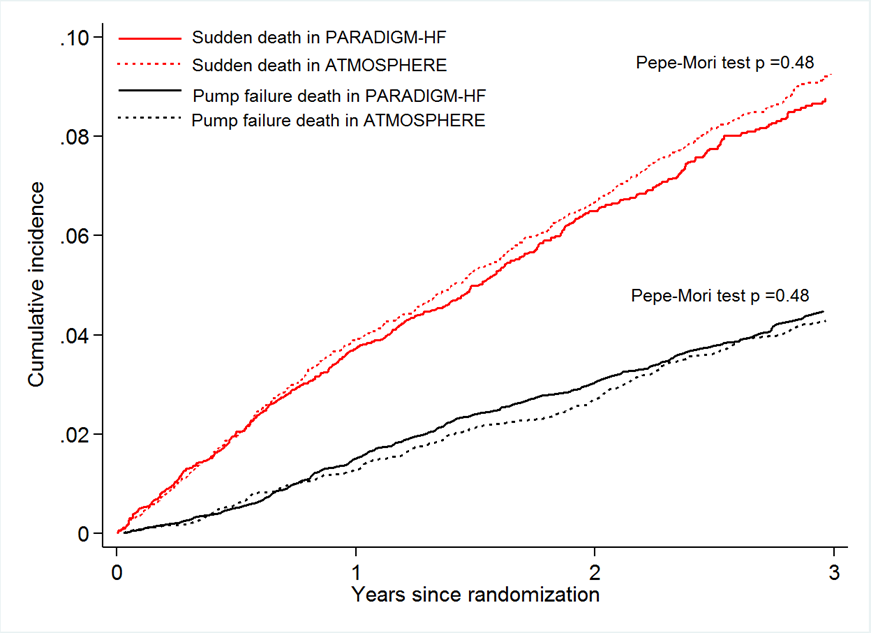


**Online Fig. A3** Validation of Seattle Proportional Risk model in PARADIGM-HF and ATMOSPHERE


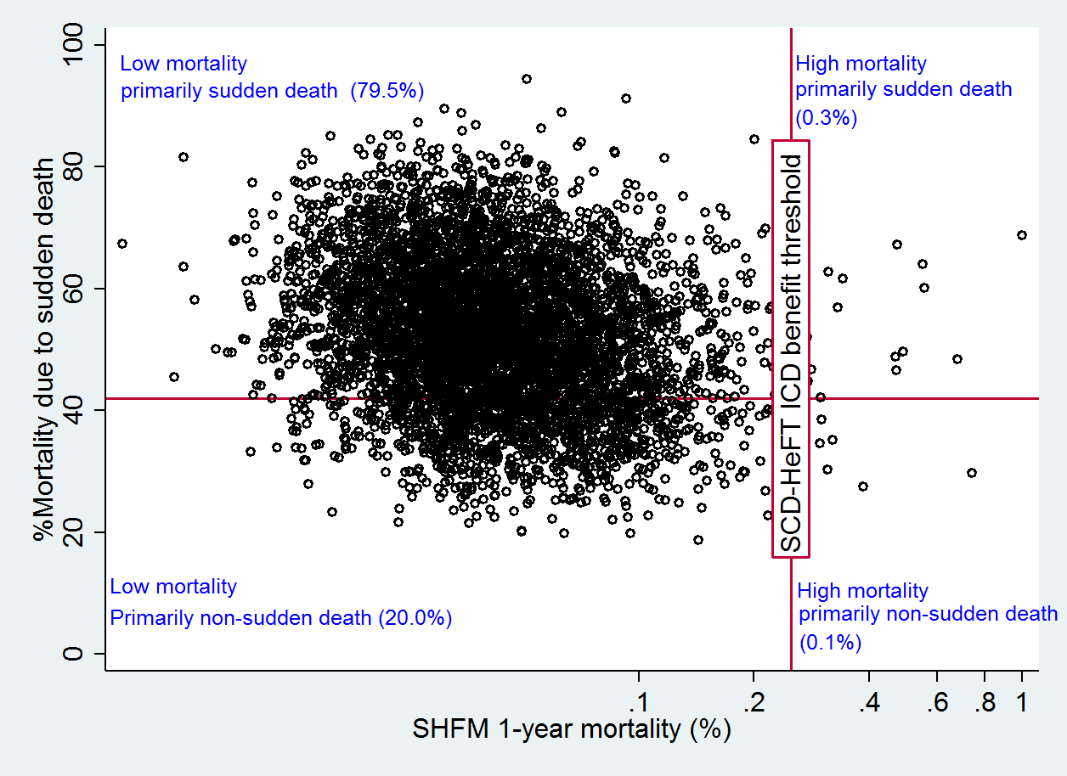

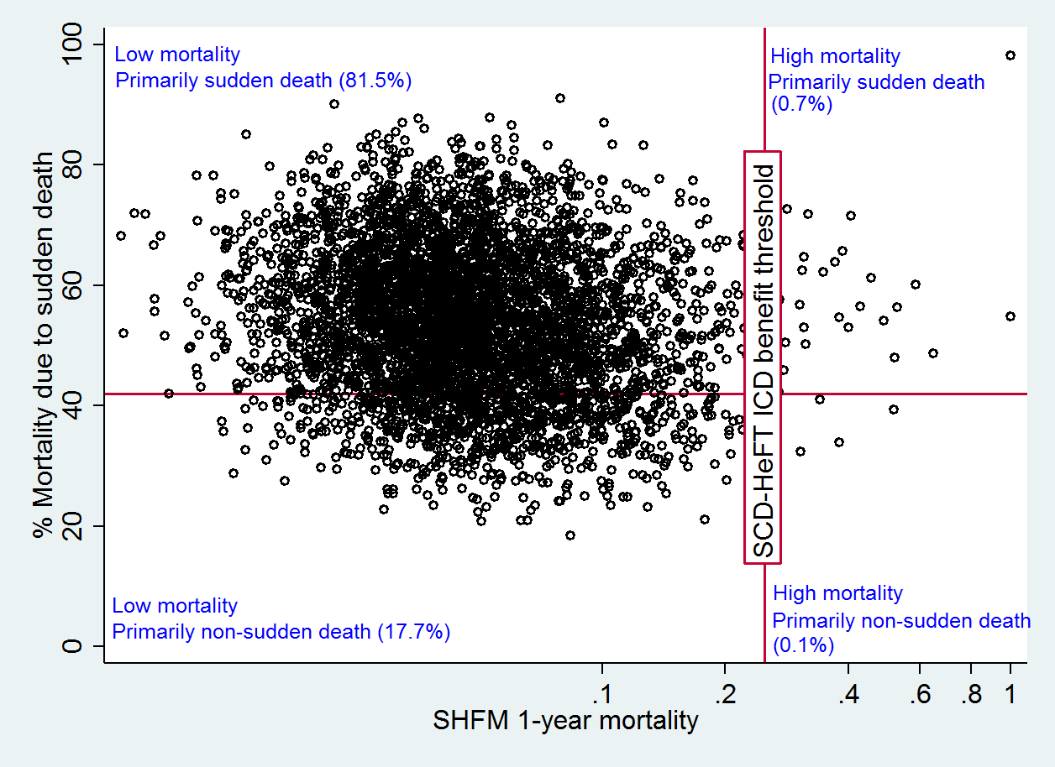


**B**

**A**

An annual all-cause mortality rate of below 25% and predicted proportion of mortality due to sudden death of over 42% were used to identify the subset of patients in the left upper quadrant who are assumed to benefit most from ICD (according to the Seattle Proportional Risk model). Data were available for calculating the risk scores of SHFM and SPRM in 5324 (74.4%) patients in PARADIGM-HF (Panel A) and 4417 (74.0%) patients in ATMOSPHERE (Panel B).

**Online Supplement** Examples of risk prediction for mode-specific death using the models presented in Table 2 and 3

Consider a white male patient, who has been diagnosed with ischemic HF for 2 years, with a LVEF 30% and NYHA class II symptoms and a systolic blood pressure 125 mmHg, had a previous PCI, a history of myocardial infraction and no other comorbidities, with a QRS duration of 110 msec and no other abnormalities on ECG, and with serum levels of albumin 40 g/L, chloride 96 mmol/L, creatinine 1.2 mg/dL, and NT-proBNP 750 pg/mL.

Based on Table 2, the risk score of sudden death is 0.361 + 0.582 -0.502 + 110/5*0.065 +ln(750)*0.348 =4.175. Figure 3A indicates that this patient is at low risk with a 4.0% probability of sudden death within 3 years.

Based on Table 3, the risk score of pump failure death is (130-125)/5*(0.096) +(40-30)*0.020 -0.372 +0.549+1.2*10*0.076 +(45-40)*(0.102) +(106-96)*(0.091) +ln(750)*0.473 = 5.936. From Figure 3B, we can see this is a low risk patient who has 2.6% chance of pump failure death within 3 years.
